# Revealing the Draft Genome Sequence of *Bradyrhizobium* sp. Strain USDA 3458, an Effective Symbiotic Diazotroph Isolated from Cowpea (*Vigna unguiculata*) Genotype IT82E-16

**DOI:** 10.1128/MRA.00813-19

**Published:** 2019-09-19

**Authors:** Richard Allen White, Jeffrey S. Norman, Emily E. Mclachlan, Joseph P. Dunham, Aaron Garoutte, Maren L. Friesen

**Affiliations:** a Department of Plant Pathology, Washington State University, Pullman, Washington, USA; b Department of Crop and Soil Sciences, Washington State University, Pullman, Washington, USA; c Department of Plant Biology, Michigan State University, East Lansing, Michigan, USA; d SeqOnce LLC, Pasadena, California, USA; e RAW Molecular Systems (RMS) LLC, Spokane, Washington, USA; f Australian Centre for Astrobiology, University of New South Wales, Sydney, NSW, Australia; University of Southern California

## Abstract

Pairing plants with plant growth-promoting bacteria is critical to the future of agriculture. Bradyrhizobium sp. strain USDA 3458 isolated from Vigna unguiculata (cowpea) paired with cowpea genotype IT82E-16 represents a novel combination in arid regions. Here, we report the draft genome sequence of strain USDA 3458.

## ANNOUNCEMENT

The rhizosphere microbiome is both numerically large and highly diverse (1–3). Critical members of the rhizosphere microbiome are symbiotic nitrogen fixers (SNF) (4). SNF account for ∼80% of all fixed nitrogen (5, 6), and SNF ability is selected for by the plant host, resulting in fitness alignment between the host and symbiont (7). Finding plant growth-promoting rhizobacteria (PGPR) has been a focus of the U.S. Department of Agriculture (USDA) for over 100 years (8, 9).

*Bradyrhizobium* sp. strain USDA 3458 was isolated from a *Vigna unguiculata* (cowpea) nodule from Nigeria in 1975 (https://data.nal.usda.gov/dataset/usda-ars-national-rhizobium-germplasm-collection). In both the greenhouse and field, experimental strain USDA 3458 formed the most effective symbiosis with the cowpea genotype IT82E-26, which is resistant to cowpea aphid (Aphis craccivora Koch) (10, 11); further inoculation with strain USDA 3458 may provide useful plant growth promotion in semiarid tropical regions (e.g., Africa).

A lyophilized culture of *Bradyrhizobium* sp. strain USDA 3458 was obtained from the Agricultural Research Service at the U.S. Department of Agriculture (USDA) National Rhizobium Germplasm Collection. The bacterial culture (single colony) was inoculated in AG broth (12) at 30°C at 200 rpm to obtain biomass for DNA extraction (12, 13).

DNA extraction, purification, and quality control are described in reference 13. Sequencing libraries were prepared using the SeqOnce RhinoSeq kit, following the manufacturer’s protocols. Sequencing was performed on a HiSeq 4000 instrument with 150-bp paired-end read format at the Michigan State University Research Technology Support Facility (RTSF).

Default parameters were used for all software, unless otherwise specified. Sequencing data were quality filtered and decontaminated using ATLAS (version 1.0) (14). The quality-controlled sequencing data (2,233,194 paired-end reads) were then assembled with Unicycler (version 0.4.7), using default Illumina assembly parameters (15).

The genome assembly contains 226 contigs, with a genome size of 8,601,019 bp, a G+C content of 63.93%, and an *N*_50_ value of 59,884 bp. CheckM (version 1.0.12) calculated strain USDA 3458 completeness to be 97.84%, with 1.04% contamination (16). We removed the genome contamination using RefineM (genomic properties setting, version 0.0.25) (17).

Prokka was used for annotation (version 1.13.3 with -rfam flag), which predicted 56 tRNAs, 1 transfer-messenger RNA (tmRNA), 37 noncoding RNAs (misc_RNA), 1 copy of a 5S-16S-23S operon, 0 CRISPRs, and 8,018 coding genes (18).

We provide this high-quality draft genome sequence as a template for further metabolic engineering and synthetic biology applications, including plant growth-promoting symbiosis in vector-resistant crops.

### Data availability.

This whole-genome shotgun project has been deposited at DDBJ/ENA/GenBank under the accession number VIDV00000000. The version described in this paper is version VIDV01000000. Raw data, contigs, and annotations for this genome can be found at https://osf.io/7t4j8/. The code used to generate the assembly can be found at https://github.com/friesenlab/Bradyrhizobium_USDA3458.
